# Four-factor prothrombin complex concentrate (Beriplex® P/N) mediated reversal of apixaban-induced bleeding in a rabbit model

**DOI:** 10.1186/cc14428

**Published:** 2015-03-16

**Authors:** E Herzog, F Kaspereit, W Krege, J Mueller-Cohrs, B Doerr, P Niebl, G Dickneite

**Affiliations:** 1 CSL Behring GmbH, Marburg, Germany

## Introduction

This study assessed whether a four-factor prothrombin complex concentrate (4F-PCC; Beriplex®/Kcentra®; CSL Behring) can effectively reverse bleeding associated with the direct oral factor Xa inhibitor apixaban in an established *in vivo *rabbit model [1,2].

## Methods

For dose-finding purposes, anesthetized rabbits were treated with a single intravenous dose of apixaban (800 to 1,600 μg/kg). In a subsequent study phase, anesthetized rabbits were treated with apixaban (1,200 μg/kg) followed by 4F-PCC (6.25 to 100 IU/kg). Bleeding signals were quantified following a standardized kidney incision by measurement of the volume of blood loss and time to hemostasis over an observation period of 30 minutes. Blood samples were collected for monitoring of coagulation parameters.

## Results

Dose-dependent increases in time to hemostasis and total blood loss were observed post apixaban administration with maximum bleeding signals seen at 1,200 μg/kg. Treatment with 4F-PCC resulted in a statistically significant reversal in apixaban-induced bleeding time (all doses) and volume (doses ≥12.5 IU/kg). Of the coagulation parameters measured, thrombin generation initiated using phospholipids only was the *in vitro *coagulation parameter most sensitive to 4F-PCC-mediated bleeding reversal, although statistically significant 4F-PCC-mediated reductions in the prothrombin time and whole blood clotting time were also observed.

## Conclusion

In conclusion, 4F-PCC treatment effectively decreased apixaban-induced hemorrhage at a clinically relevant dose range.
